# Prevalence of mental health symptoms in Austrian veterinarians and examination of influencing factors

**DOI:** 10.1038/s41598-024-64359-z

**Published:** 2024-06-10

**Authors:** Viktoria Neubauer, Rachel Dale, Thomas Probst, Christoph Pieh, Katharina Janowitz, Deianira Brühl, Elke Humer

**Affiliations:** 1 Centre for Food Science and Veterinary Public Health, Clinical Department for Farm Animals and Food System Science, University of Veterinary Medicine Vienna, Vienna, Austria; 2 FFoQSI GmbH – Austrian Competence Centre for Feed and Food Quality, Safety and Innovation, Tulln, Austria; 3 Department for Psychosomatic Medicine and Psychotherapy, University for Continuing Education Krems, Krems, Austria; 4 Division of Psychotherapy, Department of Psychology, Paris Lodron University Salzburg, Salzburg, Austria; 5 Faculty of Psychotherapy Science, Sigmund Freud University Vienna, Vienna, Austria

**Keywords:** Veterinarians, Well-being, Depression, Anxiety, Insomnia, Stress, Alcohol abuse, Psychology, Health care, Health occupations, Risk factors, Signs and symptoms

## Abstract

Although previous studies point to a high mental health burden in veterinarians, little is known about mental health in Austrian veterinarians as well as potential underlying factors of poor mental health. We assessed mental health in Austrian veterinarians, compared it to the mental health of the general population, and explored potential risk factors for poor mental health in veterinarians. A total of n = 440 veterinarians (72.0% women; mean age: 44.53 ± 11.25 years) took part in an online survey in 2022 in which validated screening tools for symptoms of depression (PHQ-9), anxiety (GAD-7), sleep disorders (ISI-2), perceived stress (PSS-4), and alcohol abuse (CAGE) were applied. Multivariable logistic regression revealed higher adjusted odds for exceeding cut-offs for clinically relevant depressive, anxiety, and insomnia symptoms in veterinarians (1.35–2.70) compared to the general population. Mental health symptoms in veterinarians were associated with female gender, physical inactivity, higher smartphone usage, higher working hours, less professional experience, and working with pets. In conclusion, it appears that veterinarians encounter mental health challenges that are more pronounced than those experienced in the general population. The teaching of strategies to improve mental hygiene as part of the curricula of veterinary education and targeted training and mentoring of employers and their team should be implemented to improve mental health in the veterinary profession.

## Introduction

Veterinarians play a crucial role in preventing, diagnosing, and curing diseases to ensure animal health. In addition, veterinarians also play an important role in safeguarding human health, due to their functions in zoonotic disease prevention and control, as well as food safety and the prevention of foodborne illnesses^1^. Their diverse responsibilities encompass various fields of work, such as general or specialized practice, education, research, diagnostic analysis, public health services, and many more. In addition to the constant commitment to further train clinically relevant knowledge and skills^1^, veterinarians also need to develop a strong ethical mindset for human–animal interactions, and respect and accommodate animal owners’ abilities, interests, and economic circumstances. Amidst these demanding professional duties, it is essential to acknowledge and address the aspect of mental health in veterinarians.

The mental health of veterinarians has been a growing concern globally, with numerous studies indicating a higher prevalence of mental health issues in this profession compared to the general population^2–7^. Previous research has highlighted the mental health burden among veterinary students in Austria, showing higher levels of depression and anxiety symptoms compared to the general population^8^. However, there is a lack of data on practicing veterinarians in Austria, who face different stressors and professional challenges. In Germany, a country with comparable living and working conditions for veterinarians as in Austria, veterinarians showed a higher prevalence of symptoms of depression and suicidal ideation than the general population^5^. Higher risk of suicide in healthcare professions compared to the general population has been reported before^9,10^, with veterinarians being often the outstanding profession. One would assume that the similar work-environment and workload of human and veterinary doctors affects their mental health similarly. However, studies indicate that veterinarians are even more likely in danger for reduced mental health than human doctors^11,12^. Recently data on suicide mortality in Austrian veterinarians were published, demonstrating higher suicide risk for veterinarians of both genders compared to the general population^13^. Compared to other male health professionals (i.e., physicians, dentists, and pharmacists), male veterinarians were the only professional group with increased suicide risk compared to the general population. We recently elaborated on Zimmermann et al.’s findings on prevalence of suicide by empirically assessing risk factors associated with higher suicidality in Austrian veterinarians^14^. These data indicate that veterinarians are a highly vulnerable group and underscore the need for further studies revealing not only suicide rates, but also a broader set of mental health indicators and factors associated with poor mental health in veterinarians, which may also assist in designing mental illness prevention campaigns.

Since the proportion of women in veterinary medicine is high and female veterinarians have a higher prevalence of mental disorders compared to male veterinarians in most studies (as reviewed by Pohl et al.^12^), gender is one of the most crucial general sociodemographic factors to consider. A higher prevalence of mental health disorders in women compared to men is not specific to the veterinary profession, but rather observed in the general population^15^. Also, associations between poor mental health and younger age, poor relationship quality, and unemployment in the Austrian general population have been reported previously^16,17^. A study conducted on a representative sample of the Austrian general population in spring 2022, further observed strong associations of health behaviors with indicators of mental health^18^. More specifically, symptoms of clinically relevant depression, anxiety, and stress levels were higher in physically inactive compared to physically active individuals. Also spending ≥ 1 h per day on a smartphone increased the odds for experiencing clinically relevant symptoms of depression, anxiety, and stress^18^.

Besides studies in the general population, different risk factors for poor mental health in veterinarians have been discussed, such as work-related stressors (i.e., long working hours, night shifts, on-call duty, working at the weekend, social isolation, dealing with clients and their expectations, and the act of performing euthanasia on their patients)^19–23^. A study conducted in Australia observed a higher prevalence of depression, anxiety, and stress in veterinarians compared to the general population, with gender, type of practice, and years after graduation being associated with the severity of mental health problems^24^.

Data on mental health among Austrian veterinarians are lacking to date. To the best of our knowledge, there was only one study carried out across German and Austrian female veterinarians, targeting their stress-management strategies^25^, but with a rather low sample size (n = 78), missing reference groups and without assessment of the perceived stress level in general.

The analysis presented here aims to offer original and valuable foundational information concerning the presence of depression, anxiety, insomnia, stress, and alcohol abuse symptoms in Austrian veterinarians. Additionally, it seeks to draw comparisons between these findings and data collected from the general population in Austria.

In order to obtain a comprehensive understanding of the factors potentially linked to poor mental health among Austrian veterinarians, we aimed to assess specific variables related to the veterinary profession (i.e., professional field, animal species, employment status, years in the profession, working hours) as well as more general variables that have been previously found to be associated with mental health in the Austrian general population (i.e., gender, age, partnership status, health behaviors)^15–18^.

Therefore, another aim of our study was to explore potential risk factors for poor mental health in veterinarians. As several risk factors are not independent of each other (e.g., higher proportion of female veterinarians working with pets), our study’s objective was not solely to assess the correlation between a single potential risk factor and mental health but also to explore the unique contribution of each independent variable in predicting the prevalence of mental health disorders, while controlling for the influence of other variables.

## Results

### Study sample characteristics

In total, n = 440 veterinarians participated (response rate: 9.70%). Table 1 provides a summary of sociodemographic and professional characteristics. They were 44.53 ± 11.25 years old, and 72.0% were female. The majority (53.9%) of the veterinarians were self-employed and had 16.81 ± 10.76 years of professional experience. They worked on average 43.95 ± 18.44 (median 40) hours per week. On average 22.02 ± 28.63 (median 10.0) hours per month were spent in nightshift or weekend work.

**Table 1:** Study sample characteristics (N = 440).

Variable	
Gender	
Female, % (N)	72.0 (317)
Male, % (N)	28.0 (123)
Age in years, M (SD)	44.53 (11.25)
Years in the profession, M (SD)	16.81 (10.76)
Working hours	
Total h per week, M (SD)	43.95 (18.44)
Night shifts and weekends h per month, M (SD)	22.02 (28.63)
Employment status	
Employed, % (N)	40.7 (179)
Self-employed, % (N)	53.9 (237)
Both, % (N)	5.5 (24)
Main professional field	
Curative practice, % (N)	85.2 (375)
University/research, % (N)	3.9 (17)
Consulting, % (N)	1.8 (8)
Abattoir, animal and meat inspection, % (N)	1.8 (8)
Official veterinarian, % (N)	5.5 (24)
Other, % (N)	1.9 (8)
Species	
Ruminants, % (N)	34.8 (153)
Pigs, % (N)	19.8 (87)
Poultry, % (N)	10.2 (45)
Horses, % (N)	37.3 (164)
Pets, % (N)	76.6 (337)
Exotic animals, % (N)	15.0 (66)

The employed subsample from the Austrian general population aged ≥ 24 years was surveyed in the same year as the sample of veterinarians and comprised n = 514 individuals. They were 43.12 ± 11.19 years old, and 48.1% were female.

Age (t(952) =  − 1.932; *P* = 0.054) did not differ between the veterinarian and the general population sample, while the veterinarians comprised more women (χ2 (1) = 56.46; *P* < 0.001).

### Mental health indicators in veterinarians vs. the general population

Chi-squared tests revealed a higher prevalence of clinically relevant symptoms of depression, anxiety, and insomnia in veterinarians compared to the general population (*P* < 0.01), while no difference was observed for clinically relevant symptoms of stress and alcohol abuse (*P* > 0.05; Table 2).

**Table 2:** Participants exceeding the cut-off scores for symptoms of clinically relevant depression, anxiety, insomnia, and stress by group (n = 954).

Variable	Group		*Statistics*
	General population (n = 514)	Veterinarians (n = 440)	
Depression			
%	25.7	33.9	χ^2^ (1) = 7.64
n	132	149	*P* = 0.006
Anxiety			
%	15.8	34.5	χ^2^ (1) = 45.33
n	81	152	*P* < 0.001
Insomnia			
%	8.0	14.3	χ^2^ (1) = 9.82
n	41	63	*P* = 0.002
High stress			
%	56.2	60.2	χ^2^ (1) = 1.56
n	289	265	*P* = 0.212
Alcohol abuse			
%	18.9	14.3	χ^2^ (1) = 3.52
n	97	63	*P* = 0.061

*P*: *P* -values (two-tailed); χ^2^: Chi-squared-test; depression: ≥ 10 points on the patient health questionnaire 9 scale; anxiety: ≥ 10 points on the generalised anxiety disorder 7 scale; insomnia: ≥ 6 on the two-item insomnia severity index; high stress: ≥ 6 points on the perceived stress scale 4; alcohol abuse: ≥ 2 on the four-item CAGE questionnaire.

**Figure 1:**
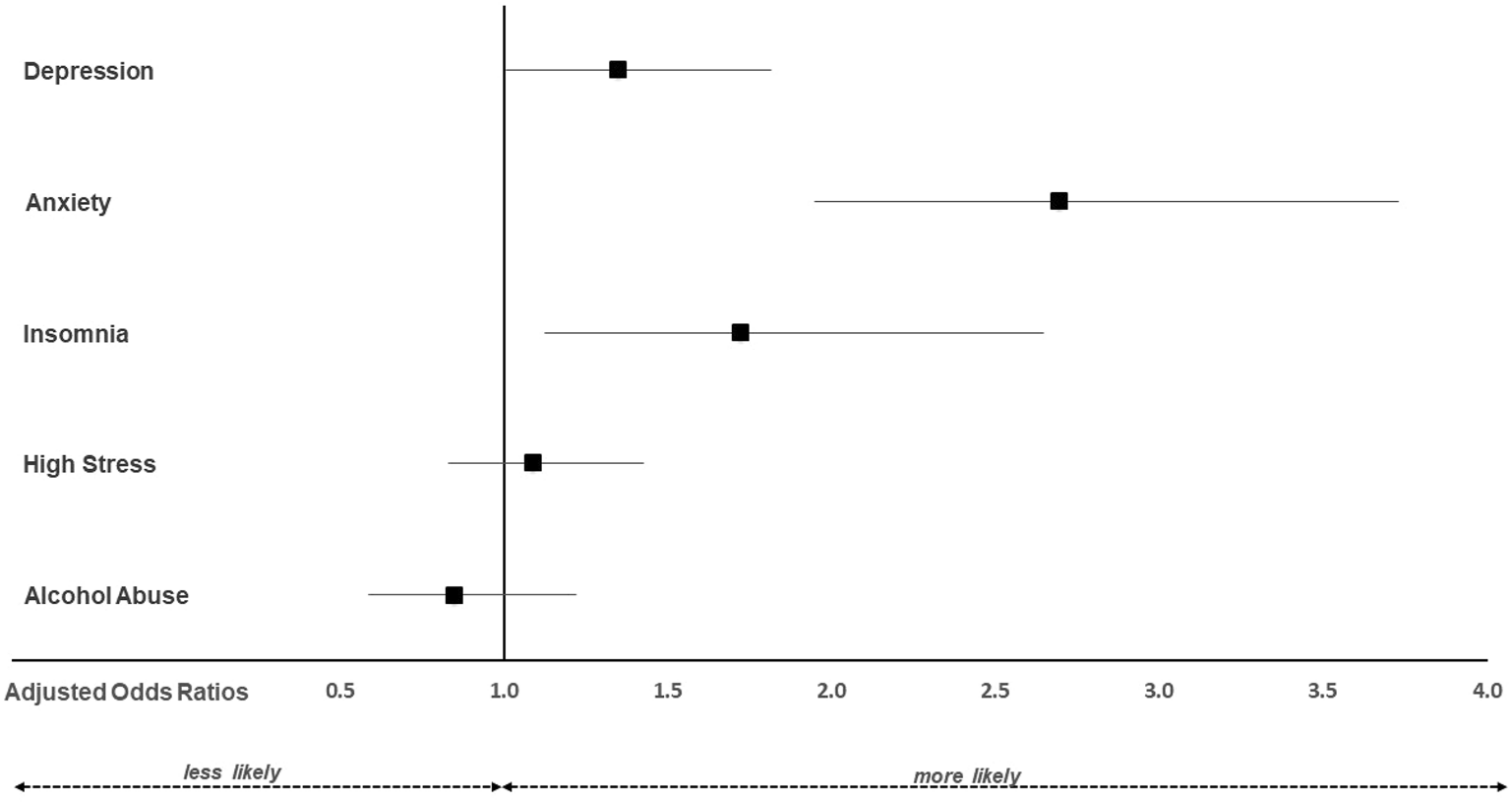
Adjusted odds ratios for clinically relevant symptoms of depression, anxiety, insomnia, stress, and alcohol abuse in veterinarians (n = 440) vs. the employed subgroup of the general population (n = 514). The multivariable model was adjusted for age and gender. An adjusted odds ratio of 1 indicates no difference. Adjusted odds ratios > 1 indicate higher relative risk in veterinarians compared to the general population. Confidence intervals (horizontal lines) crossing 1 (vertical line) indicate no significant difference between veterinarians and the general population.

Including age and gender in the multivariable logistic regression analyses confirmed findings of the univariate analyses. As depicted in Fig. 1, veterinarians, compared to the general population, were more likely to experience clinically relevant depression (aOR 1.35; 95% CI 1.01, 1.81), anxiety (aOR 2.70; 95% CI 1.95, 3.73), and insomnia (aOR 1.72; 95% CI 1.12, 2.65). The adjusted odds for high-stress levels (aOR 1.09; 95% CI 0.83, 1.43) and alcohol abuse (aOR 0.85; 95% CI 0.59, 1.22) did not differ between groups.

In the total study group (veterinarians and general population) female gender increased the odds for depression (aOR 1.76; 95% CI 1.29, 2.41), anxiety (aOR 1.60; 95% CI 1.14, 2.26), insomnia (aOR 1.62; 95% CI 1.02, 2.58), and high stress (aOR 1.57; 95% CI 1.19, 2.06), whereas it decreased the odds for alcohol abuse (aOR 0.61; 95% CI 0.43, 0.88). With increasing age, the odds for depression (aOR 0.97; 95% CI 0.96, 0.99), anxiety (aOR 0.97; 95% CI 0.96, 0.98), high stress (aOR 0.98; 95% CI 0.97, 0.99), and alcohol abuse (aOR 0.97; 95% CI 0.96, 0.99) decreased, whereas for insomnia no significant age effect was observed (aOR 1.01; 95% CI 0.99, 1.02).

### Association of sociodemographic factors, health behaviors, and work-related variables with mental health indicators in veterinarians

#### Sociodemographic variables

Female gender in veterinarians was associated with higher odds for clinically relevant symptoms of depression (aOR 1.84; 95% CI 1.02; 3.30). Increased odds for clinically relevant symptoms of insomnia with increasing age (aOR 1.08; 95% CI 1.01; 1.15) were observed. Partnership status was not associated with mental health indicators in veterinarians.

#### Health behaviors

**Figure 2:**
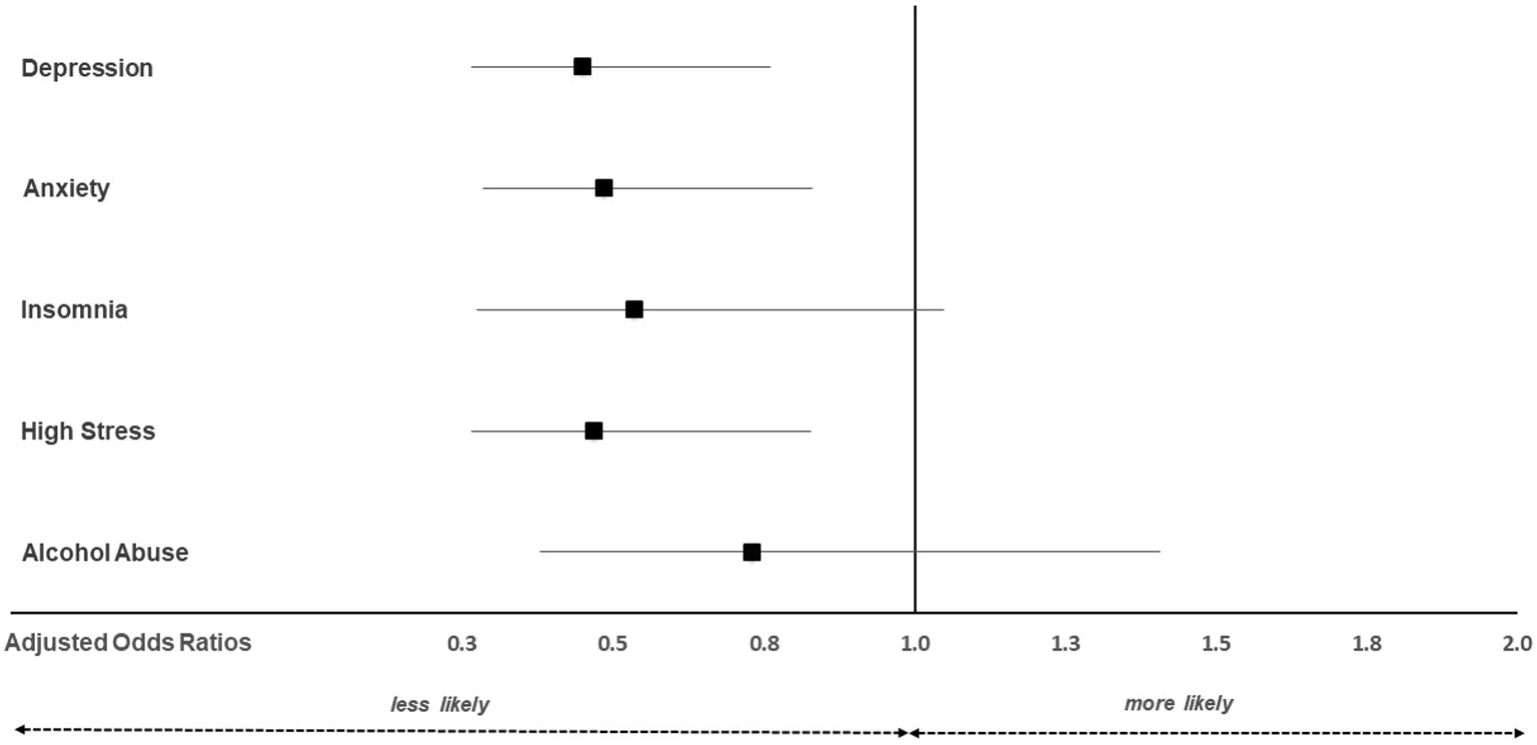
Adjusted odds ratios for clinically relevant symptoms of depression, anxiety, insomnia, stress, and alcohol abuse in physically active (≥ 1 day/week physically active outside of the job; n = 358) vs. inactive (< 1 day/week physically active outside of the job; n = 82) veterinarians. The multivariable model was adjusted for age, gender, partnership status, smartphone usage, years in the profession, workload, work spent in nightshifts/at weekends, employment status, professional field, and working with ruminants, pigs, horses, poultry, pets, or exotic animals. An adjusted odds ratio of 1 indicates no difference. Adjusted odds ratios < 1 indicate lower relative risk in physically active veterinarians compared to physically inactive veterinarians. Confidence intervals (horizontal lines) crossing 1 (vertical line) indicate no significant difference between physically active and inactive veterinarians.

Physical activity (defined as being physically active outside of professional activities for at least 60 min per day on at least 1 day per week) was associated with reduced risk for exceeding the cut-offs for clinically relevant symptoms of depression (aOR 0.45; 95% CI 0.27; 0.76), anxiety (aOR 0.49; 95% CI 0.29; 0.83), and stress (aOR 0.47; 95% CI 0.27; 0.83) as depicted in Fig. 2.

**Figure 3:**
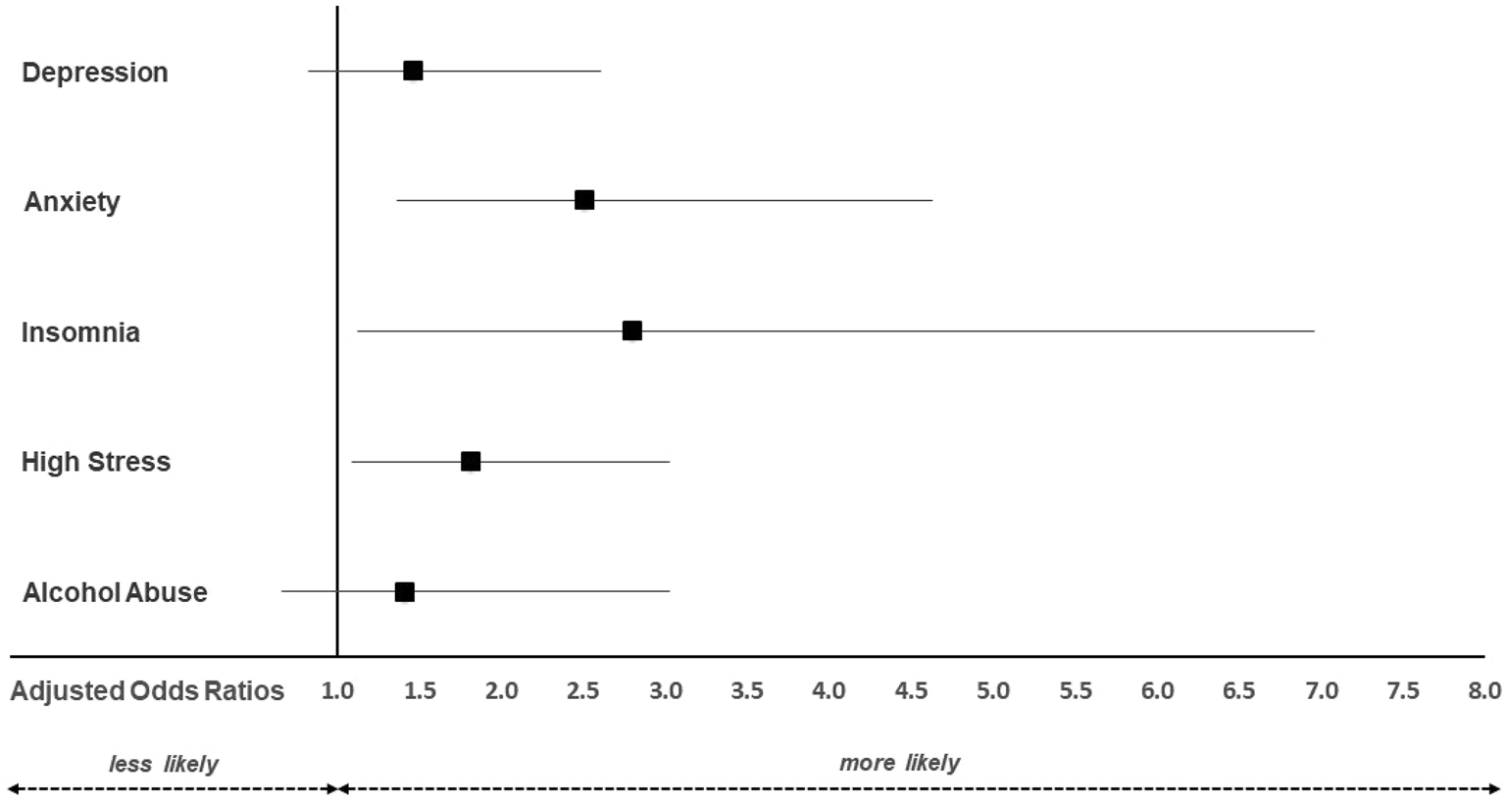
Adjusted odds ratios for clinically relevant symptoms of depression, anxiety, insomnia, stress, and alcohol abuse in veterinarians using the smartphone for ≥ 1 h per day (n = 351) vs. < 1 h per day (n = 89). The multivariable model was adjusted for gender, age, partnership status, physical activity outside the of the job, years in the profession, workload, work spent in nightshifts/at weekends, employment status, professional field, and working with ruminants, pigs, horses, poultry, pets, or exotic animals. An adjusted odds ratio of 1 indicates no difference. Adjusted odds ratios > 1 indicate higher relative risk in veterinarians spending at least 1 h/d on their smartphones compared to those spending up to 1 h/day on the smartphones. Confidence intervals (horizontal lines) crossing 1 (vertical line) indicate no significant difference between physically active and inactive veterinarians.

Using a smartphone for at least 1 h per day increased the adjusted odds of experiencing clinically relevant symptoms of anxiety (aOR 2.51; 95% CI 1.36; 4.62), insomnia (aOR 2.80; 95% CI 1.13; 6.95), and stress (aOR 1.82; 95% CI 1.09; 3.03) in veterinarians (Fig. 3).

#### Work-related variables

Among the variables related to professional activities (years in profession, workload, work spent in nightshifts/at weekends, employment status, professional field, animal species), a higher workload was found to be associated with increased odds for clinically relevant symptoms of depression (aOR 1.02; 95% CI 1.00, 1.03) and anxiety (aOR 1.02; 95% CI 1.00, 1.03). Being in the profession for a longer period decreased the odds of high stress symptoms (aOR 0.94; 95% CI 0.88; 0.995). No associations of mental health indicators with work spent on nightshifts/at weekends, employment status, and professional field were found. Among animal species, only working with pets was associated with increased odds for experiencing symptoms of clinically relevant anxiety (aOR 2.10; 95% CI 1.20, 3.68) and stress (aOR 1.70; 95% CI 1.02, 2.83).

## Discussion

### Mental health in veterinarians compared to the general population

This study builds on the findings of our previous research on Austrian veterinary students^8^ by examining a different but related population: practicing veterinarians. While both groups exhibit higher levels of mental health symptoms compared to the general population, there are differences in the factors influencing their mental health due to variations in professional responsibilities, work environments, and life stages. Our findings suggest that practicing veterinarians face more pronounced mental health challenges compared to the employed subgroup of the general population. Veterinarians exhibited significantly higher odds (ranging from 1.35 to 2.70) of surpassing the threshold for clinically relevant symptoms of depression, anxiety, and insomnia in comparison to the general population. These results highlight the need for targeted mental health interventions tailored to the unique needs of veterinarians at different stages of their careers.

Similarly, higher levels of depression, anxiety, and stress were observed in Australian veterinarians compared to the general population^24^. Results on higher risk for insomnia in comparison to the general population are supported by a recent study conducted among US veterinarians, showing higher odds of trouble sleeping in veterinarians even compared with various reference groups, including physicians and dentists^11^.

A previous study conducted on German veterinarians in 2016 observed clinically relevant symptoms of depression, assessed with the same instrument (PHQ-9), in 28% of the veterinarians, but only in 4% of the general population^5^. The current study observed a higher prevalence in veterinarians (34%) and in the general population (26%). The large difference in the general populations between the German study in 2016 and the current study in 2022 could be attributed to a general increase in depression since the COVID-19 pandemic^15,16^. Studies on mental health in veterinarians during the COVID-19 pandemic are limited, with some data pointing at lower well-being, particularly in furloughed veterinarians^26^. Summarizing these findings, although mental health of veterinarians remains worse than the general public, it is possible that the general population suffered mentally more during and post COVID pandemic (increase from 4 to 26%) than the veterinary profession (increase from 28 to 34%). It can be speculated that being attributed as one of the critical professions during the pandemic might have prevented veterinarians from greater impairment in mental health compared to the general population. Being part of the critical infrastructure, veterinarians in Austria never faced full lockdowns and could continue their businesses. Another reason could have been that health professions were already used to hygienic measures, like wearing masks or disinfecting hands, and are aware of disease-prevention necessities, like quarantine and vaccination. Nonetheless, additional analyses are required to elucidate the impact of the pandemic on the mental health of veterinarians. Additionally, the impact of the pandemic on the mental health of veterinarians may vary across different career stages. This is especially true for recently graduated veterinarians who did part of their veterinary education during the pandemic. Their experiences and coping mechanisms could differ significantly from those who graduated before the pandemic or had more established practices. Considering the unique challenges and stressors faced by this specific group, further research focusing on the mental well-being of recently graduated veterinarians could provide valuable insights into the long-term effects of the pandemic on the veterinary profession.

### Sociodemographic, health behavior, and work-related factors associated with poor mental health

A further main finding of the study was that mental health problems in veterinarians were associated with female gender, physical inactivity outside of professional work, higher time spent on a smartphone, higher working hours, fewer years in the profession, and working with pets.

The findings regarding the heightened mental health burden in women versus men as well as in physically inactive individuals and those spending more time with their smartphones, mirror patterns observed in the general population^16,18^. Gender differences in mental health problems were well-known already before the pandemic, with higher rates of depression and anxiety in women and higher rates of substance abuse and antisocial disorders in men^27^. Similarly, the positive effects of physical activity are well-documented. Due to the stress-reducing and antidepressant effect of physical activity^28,29^, the current findings emphasize the importance of encouraging physical activity that is not related to work in veterinarians to mitigate mental health problems. Also, the association between excessive smartphone usage and poor mental health has been reported in several previous studies^18,30^ and highlights the need to keep smartphone usage at a low level (< 1 h/day) to promote mental health. The odds of experiencing high stress levels were associated with fewer years in the profession. Psychosocial stressors at work are well-documented contributors to mental disorders. A recent systematic review and meta-analysis revealed that encountering psychosocial work-related stressors is associated with a heightened risk of sickness absences due to diagnosed mental disorders^31^. The proportions of sickness absence and early retirement due to mental health issues have risen across Europe over the past few decades^32^. A recent study, conducted in 2022/2023 on European veterinarians^33^, revealed that 23% of veterinarians reported more than 2 weeks off-work due to burnout, exhaustion, compassion fatigue, or depression within the past 3 years. These percentages varied greatly among countries, with Luxembourg and Hungary reporting lower rates, while Latvia and North Macedonia reported higher rates. The rate reported by the n = 278 participating Austrian veterinarians was below the average (14%). Female and early-career veterinarians consistently reported higher rates of medical leave compared to male and senior veterinarians. Pohl et al.^12^ summarized in their review that newly qualified veterinarians have a higher risk of suffering occupational stress and are more demoralized than those well-established in the profession. Nett et al.^3^ found a negative association of years in practice or age in US veterinarians with severe psychological distress, but no clear association between depression and suicidal ideation with years in the profession or age. Several other studies conducted on veterinarians in Australia, Finland, and New Zealand observed increased mental health burdens in more recent graduates^24,34,35^. Potential reasons might be the uncertainty regarding professional knowledge and skills, difficulties in transferring the obtained theoretical knowledge into practice, and lack of mentoring ^24^. Another factor might be the lower development of skills for coping due to the lower work experience during the first years after graduation. It is also possible that practitioners who struggle with practical work might have left the profession and might therefore not be represented in the sample of veterinarians who graduated in more recent years^36^. The Austrian graduate-tracking survey (ATRACK) reported that 3 years after graduation from the University of Veterinary Medicine Vienna, Austria (graduation years 2008/09–2020/21), 30% were not working in the veterinary profession^37^. Data from Australia report that 20% of veterinary students were no longer in veterinary practice 10 years after graduation^36^. A survey of the veterinary profession in Europe conducted between November 2018 and March 2019^38^, observed that 27% of the n = 212 participating Austrian veterinarians considered leaving the veterinary sector in less than 5 years. This proportion is below the average of 32% observed across the total of 30 surveyed European countries. Overall, the potential interest in transitioning to a non-veterinary occupation exhibited significant variability among countries, ranging from less than 20% in Hungary, Luxembourg, France and Belgium to over 40% in Italy, Serbia, Iceland, Russia and Portugal^38^.

Among work-related variables, the workload, assessed as hours spent working per week, was also associated with the investigated mental health indicators. The adjusted odds for symptoms of clinically relevant depression and anxiety were associated with increasing hours of work. Studies conducted in Finland, Australia, and New Zealand observed that the hours worked represented one of the main stressors in the veterinary profession^34–36^. Also, Pohl et al.^12^ highlight in their recent review that the more hours the veterinarians worked, the more stressed they were. Among potential stress sources, the high demands of practice (i.e., long working hours, on-call duty) have been listed as the most prominent stress factors reported by practice owners, practice associates, and relief vets^3^. Poor work-life balance was also reported to be the top reason to leave the veterinary profession^12^. Thus, measures to improve work-life balance, focusing on a reduction of working hours or on-call duties, promoting non-job-related physical activity, and professional support and mentoring for psychological distress might be a starting point to reduce stress levels and mental health burdens in the veterinary profession.

In our study, no association between the professional field (in curative practice vs. not in curative practice) and mental health symptoms has been observed. However, veterinarians treating pets showed higher odds of experiencing clinically relevant anxiety and stress symptoms. Previous findings concerning the relationship between the type of veterinary practice and mental health have generated conflicting results^24,36^. Hansez et al.^39^ found higher job strain in small animal veterinarians in comparison to mixed practices, but no difference in comparison to bovine practice. Nett et al.^3^ also found higher serious psychological distress for vets who work in shelters, which assumedly host mainly small animals, higher previous depression for small animal and shelter vets, and higher suicidal ideation for small, shelter and exotic animals. In a study conducted on Australian veterinary surgeons, high levels of psychological distress and compassion fatigue were observed. One of the key factors impacting the mental health of veterinarians was found to be the dealing with clients who were grieving the loss of their companion animals^40^. As small animal practitioners seem to be the ones at the highest risk for poor mental health, further in-depth analyses are needed to elucidate the specific stress factors associated with working with pets to enable the provision of specific workplace health promotion strategies.

### Limitations

This study has several limitations. The cross-sectional design of the study prevents the establishment of causal relationships. All mental health measures relied on self-reported data and were not verified through structured or standardized clinical interviews, primarily due to the online format of the study. Notably, no differentiation between various smartphone activities was possible, potentially impacting mental health outcomes differently. While smartphone use exceeding one hour was recorded, the survey did not capture the specific nature of this usage, suggesting the need for future research with more refined measures. The online data collection may introduce selection bias, potentially affecting the reported mental health status of Austrian veterinarians. Whether individuals experiencing significant psychological distress were inclined or disinclined to participate in the questionnaire remains unclear, as it is uncertain whether they were motivated by their heightened interest in the topic or discouraged by factors such as reduced interest or energy. This introduces uncertainty regarding the generalizability of our findings to the broader population of Austrian veterinarians. To minimize selection bias, all practicing veterinarians in Austria were invited to participate in the online survey through the Austrian Chamber of Veterinarians. Additionally, statistical analyses were adjusted for potential confounding variables, including gender and age. Despite these efforts, the influence of response bias on our findings should be recognized when interpreting the results. Moreover, low response rates (6.2% in the general population, 9.7% in the veterinary sample) could further exacerbate selection bias and limit statistical power, possibly skewing results and increasing the type II error risk. While samples were controlled for age and gender, they were not adjusted for variations in region and income. Moderate internal consistency of the CAGE in both veterinarians and the general population samples poses another limitation. Furthermore, due to the cross-sectional design, an already high rate of mental health disorders upon deciding to work in the veterinary field cannot be ruled out. Indeed, the individual personality can have a higher prediction level for job-related stress than the job environment^41,42^. We need to consider that persons who study veterinary medicine, have specific cognitive, and personality characteristics including specific attitudes to death and euthanasia^43^. For instance, it has been observed that mental health professionals are more inclined to have early experience of childhood trauma and family dysfunction compared to individuals in other occupations^44^. It is plausible that the decision to pursue a veterinary career might be subconsciously shaped by factors such as a preference for working with animals over people, potentially leading to relative social isolation, which in turn could impact the risk of depressive illnesses. The veterinary education, however, might inhibit social and communication skills due to its high demand for study hours, and competitiveness, even more than in human medicine^20^.

## Conclusion

Results suggest that poor mental health is quite common among Austrian veterinarians. Austrian veterinarians seem to experience poorer mental well-being than the general Austrian population. Additional research is warranted to provide a more comprehensive understanding of the underlying detrimental factors behind these findings. Professional organizations and veterinary educational institutions may want to contemplate the advantages of enhancing the curriculum to better equip veterinarians in managing work-related distress, anxiety, and depression, with the goal of enhancing mental well-being and potentially reducing attrition from the profession.

## Methods

### Design

An online survey among licensed Austrian veterinarians was conducted between November 16 and December 19, 2022. The link to the survey was sent via e-mail to all veterinarians registered in the list of the Austrian Chamber of Veterinarians who provided a valid e-mail address (4534 veterinarians). Veterinarians’ participation was voluntary, without incentives. From the invited veterinarians, 9.7% (n = 440) participated and answered all questions. A total of n = 608 individuals clicked on the survey link, resulting in a participation rate (i.e., completion rate) of 72.4%.

In the same year (April 2022), a representative sample of the Austrian general population was recruited as described in detail previously^15^. In brief, a core online access panel with 130,000 survey participants in Austria was surveyed via a quota sampling approach to ensure representation across critical demographics, including age, gender, age-gender combinations, region, and educational level. When the quota for the total sample (approx. 1000) was reached, the survey was closed. From the invited panelists 6.2% responded to the survey. The precise comparison for the study population (n = 1031) of the intended quota (based on the data from the Austrian Federal Statistics Office) compared to the reached quota for this online survey is given in the supplementary materials of Humer et al.^15^. Only employed participants aged ≥ 24 years (n = 514) were used as a general population comparison group, as the youngest participants in the veterinarians’ sample were 24 years old and only veterinarians currently working in the profession were included in the statistical analysis. Both surveys were carried out during the third year of the COVID-19 pandemic. As Austria lifted most containment efforts in the spring of 2022^45,46^, both surveys were carried out during a period with only minimal restrictions in place.

This study was carried out in accordance with the principles outlined in the Declaration of Helsinki and received approval from the Ethics Committee of the University for continuing education Krems, Austria (Ethical number: EK GZ 25/2021–2024). All participants provided electronic informed consent to engage in the study upon completing the questionnaires.

### Measures

#### Sociodemographic variables and health behaviors

All participants were asked about their gender (man, woman, diverse), age (in years), and partnership status (single, in partnership). Veterinarians were further asked about their years in the profession, working hours (per week), working hours in the form of night shifts and weekends (per month), the animal species they are working with (ruminants, pigs, horses, poultry, pets, exotic animals), as well as their employment status (employed, self-employed) and professional field (curative practice; university/research; consulting; abattoir, animal, and meat inspection; official veterinarian). Health behaviors (smartphone use, physical activity) were assessed by self-report. Physical activity was assessed by asking on how many of the last 7 days participants were physically active for at least 60 min per day. To elucidate physical activity outside the professional work, this question was asked a second time asking on how many of the last 7 days participants were physically active for at least 60 min per day outside of their job. Smartphone use was assessed also by self-report, the participants were asked about the time they spend in a typical day on their smartphone. The response choices for this question included the following time intervals: less than 1 h per day, 1–2 h per day, 3–4 h per day, 5–6 h per day, 7–8 h per day, and more than 8 h per day.

#### Perceived stress (PSS-4)

The assessment of perceived stress levels was conducted using the perceived stress scale (PSS-4), a self-report tool comprising four items. Respondents rated their stress levels on a five-point Likert scale, ranging from 0 (never) to 4 (very often)^47^. Notably, items 2 and 3 are reversed-coded. The total PSS-4 scores varied from 0 to 16, with higher scores indicating a higher perception of stress. A stress score of ≥ 6 was considered indicative of high stress levels^48^. Cronbach’s alpha for internal consistency was α = 0.83 in the present veterinarian sample, while in the general population subsample it was α = 0.71.

#### Depressive symptoms (PHQ-9)

The depression module of the patient health questionnaire was applied to measure depressive symptoms^49^. The PHQ-9 comprises nine self-rating items designed to assess symptoms of depression experienced over the preceding 2 weeks. Respondents provide ratings on a four-point scale, with 0 signifying “not at all” and 3 indicating “nearly every day”, yielding a total score from 0 to 27, with a cut-off point of at least 10 points indicating moderate (clinically relevant) depressive symptoms^50^. Cronbach’s alpha was α = 0.86 in the present veterinarian sample and α = 0.88 in the general population subsample.

#### Anxiety (GAD-7)

Anxiety symptoms were assessed with the seven self-rating items of the generalized anxiety disorder 7 scale^51^. The GAD-7 measures symptoms of generalized anxiety over the last 2 weeks on a four-point scale from 0 (not at all) to 3 (nearly every day). Total scores range from 0 to 21, with scores of ≥ 10 indicating clinically relevant anxiety symptoms^52^. Cronbach’s alpha was α = 0.86 in the present veterinarian sample and α = 0.89 in the general population subsample.

#### Insomnia (ISI-2)

The assessment of sleep quality was conducted using the two-item version of the insomnia severity index (ISI)^53^. The self-rating items of the ISI-2 gauge an individual’s satisfaction or dissatisfaction with their current sleep patterns and the degree to which these patterns interfere with daily functioning. These items are rated on a five-point Likert scale, ranging from 0 to 4. The total score on the ISI-2 can range from 0 to 8. A cut-off score of ≥ 6 has been suggested to indicate insomnia disorder ^54^. Cronbach.s alpha was α = 0.71 in the present veterinarian sample and α = 0.72 in the subsample of the general population.

#### Alcohol abuse (CAGE)

The CAGE questionnaire was applied to assess symptoms of alcohol abuse^55^. The four yes/no questions of the CAGE ask about signs of alcoholism (questions about cutting down, annoyance with criticism, feelings of guilt, and eye-openers). Total scores range from 0 to 4, with scores ≥ 2 indicating alcohol abuse^56^. Cronbach’s alpha was α = 0.57 in the present veterinarian sample and α = 0.67 in the subsample of the general population.

### Statistical analyses

Descriptive statistics were utilized to summarize sociodemographic characteristics. To examine variations in sociodemographic attributes between the participating veterinarians and the subsample of the Austrian general population, Chi-squared tests and *t*-tests for independent samples were employed.

Chi-squared tests were conducted to analyze differences in the prevalence of clinically relevant symptoms of depression, anxiety, insomnia, stress, and alcohol abuse between veterinarians and the general population.

Multivariable binary logistic regression analysis was employed to control for potential confounding variables when comparing both groups (veterinarians vs. general population) by including age and gender as predictors. The dichotomized mental health variables (clinically relevant vs. not clinically relevant) were included as dependent variables and the group (veterinarians vs. general population), gender (female vs. male), and age as predictors. Additionally, potential interactions between gender and group were tested, but no significant findings were observed, thus this interaction was removed from the final models. Adjusted odds ratios (aOR) along with their corresponding 95% confidence intervals (CIs) were calculated to evaluate the statistical uncertainty.

To delve deeper into the factors linked to clinically relevant mental health symptoms within the veterinarian sample, multivariable binary logistic regression was used with the mental health variables as dependent variables and gender (female vs. male), age, partnership status (single vs. in partnership), physical activity (≥ 1 day/week vs. < 1 day/week physically active outside of the job), smartphone usage (≥ 1 h/day vs. < 1 h/day), years in the profession, workload (hours/week), work spent in nightshifts/at weekends (hours/month), employment status (self-employed vs. employed), professional field (in curative practice vs. not in curative practice), and working with ruminants (yes vs. no), pigs (yes vs. no), horses (yes vs. no), poultry (yes vs. no), pets (yes vs. no), or exotic animals (yes vs. no) as predictors.

Statistical analyses were carried out using SPSS version 26, developed by IBM Corp in Armonk, NY, USA. Significance levels were set at *P* < 0.05 for all statistical tests, using two-sided assessments.

## Data Availability

The datasets used and analyzed during the current study are available from the corresponding author on reasonable request.
